# Individual Differences in Sound-in-Noise Perception Are Related to the Strength of Short-Latency Neural Responses to Noise

**DOI:** 10.1371/journal.pone.0017266

**Published:** 2011-02-28

**Authors:** Ekaterina Vinnik, Pavel M. Itskov, Evan Balaban

**Affiliations:** 1 Cognitive Neuroscience Sector, SISSA, Trieste, Italy; 2 Behavioral Neurosciences Program, McGill University, Montreal, Quebec, Canada; Duke University, United States of America

## Abstract

Important sounds can be easily missed or misidentified in the presence of extraneous noise. We describe an auditory illusion in which a continuous ongoing tone becomes inaudible during a brief, non-masking noise burst more than one octave away, which is unexpected given the frequency resolution of human hearing. Participants strongly susceptible to this illusory discontinuity did not perceive illusory auditory continuity (in which a sound subjectively continues during a burst of masking noise) when the noises were short, yet did so at longer noise durations. Participants who were not prone to illusory discontinuity showed robust early electroencephalographic responses at 40–66 ms after noise burst onset, whereas those prone to the illusion lacked these early responses. These data suggest that short-latency neural responses to auditory scene components reflect subsequent individual differences in the parsing of auditory scenes.

## Introduction

Natural and urban environments are full of unpredictable noises that interrupt meaningful sounds - a major problem that the auditory system needs to cope with. Auditory stream segregation [1], [2], perceptual restoration [3], [4] and selective attention are among the most important perceptual processes that are recruited to help in this task.

Segregation based on frequency, timbre and comodulation patterns allows listeners to treat sounds that potentially come from different objects as dynamically separable auditory sources (such as a voice from a single person that emerges out of the background din at a cocktail party, or independent melodic lines associated with different instruments in an orchestra performance). If one of the sounds in a scene is weak and another is so loud that it completely obscures the weaker sound, the peripheral neural representation of the mixture is dominated by the stronger ‘masking’ sound to such a degree that it may not be possible to reliably extract information about the weaker sound. Auditory systems cope with this type of masking by using context or previous knowledge to “fill in” the missing information [5]. The results of this filling-in operation can be so convincing that even a discontinuous sound will be perceived as continuous if the gap is filled with a louder masking noise, a phenomenon known as the continuity illusion (reviewed in [5]). Perceived continuity not only allows listeners to keep better track of sounds as they unfold over time, but can also aid sound recognition, as in the case of speech in noise [6], [7].

Previous authors have drawn a connection between continuity illusions and sound source segregation (reviewed in [2], p.369, see also [8]). Because auditory streaming happens both in the presence and absence of perceptual restoration [9], it has been argued that a putative grouping mechanism that links acoustically similar parts of an auditory scene together into single objects (segregated from other such objects) also underlies continuity illusions [2]. In line with this hypothesis, a single computational model was able to account for both auditory streaming and continuity illusions [10]. While a number of physiological studies have begun to explore underlying neural mechanisms [11]–[22], a detailed understanding of how auditory source segregation and perceptual restoration are performed in the brain is still lacking. Correspondingly, we know little about the physiological factors that influence individual variation in the sound-in-noise performance of normally-hearing individuals.

Healthy young adults with normal hearing thresholds can differ substantially in their individual abilities to detect target sounds in the presence of spectrally dissimilar irrelevant sounds. Their performance is typically worse if the maskers are unpredictable (changing from trial to trial); thresholds in the presence of such maskers can differ among individual, normally-hearing listeners by up to 59 dB [23]–[25]. The performance of the listeners who are most affected by the distracters is not easily improved by training, suggesting a possible basis in auditory system function rather than listening strategy.

Listeners who perform differently in detecting target sounds in the presence of spectrally-dissimilar noise maskers (informational masking) do not have systematic differences in frequency selectivity when measured using the notched-noise method [26], [27]. It has been proposed that a listener's ability to detect signals in the presence of extraneous sounds depends on the way in which they integrate information over a larger number of auditory filters, or on their converse ability to segregate information coming from each auditory filter [24], [26], [28].

The present research project began with a puzzling phenomenon we encountered during pilot experiments designed to investigate auditory continuity illusions. Participants were instructed to ignore distracting noises accompanying target tones, and to judge the tones as continuous or discontinuous. They were presented with training examples of sounds that included continuous tones that were accompanied by a brief noise burst more than an octave away that came on and off in the middle of the tone. Several participants were insistent that these tones sounded discontinuous to them. More detailed data collection confirmed that those participants not only reliably judged these continuous sounds as discontinuous, but also did not perceive continuity illusions at short noise durations, where previous work had shown that these illusions tended to be the strongest [5], [29].

We hypothesized that these individuals' failure to maintain perceptual continuity of the tone was due to some kind of interference between the neural representations of tone and the noise, leading to difficulty attending to the tone during the noise. To further examine these ideas, we characterized the behavior of 46 normally-hearing young adult participants (Experiment 1), and found that 24% of them consistently perceived illusory discontinuity. Experiment 2 used EEG to characterize evoked responses to tones-in-noise and noise bursts alone, in an attempt to differentiate between possible mechanisms underlying perceptual interference. To minimize any confounds due to volitional attention, the sounds were presented in a passive listening paradigm, while participants watched an engaging silent movie of their choice; participants were also instructed to ignore the sounds.

## Methods

All procedures were approved by the SISSA BioEthics Committee. Fifty-four participants provided written informed consent before participating in the experiments. The participants had no history of peripheral or central hearing disorders, and had their hearing levels characterized. Data from 8 of these subjects were not considered for further analysis (1 because of a large interaural hearing level difference, 1 because of poor performance in catch trials in the psychophysical tasks, and 6 who were unavailable to complete all of the data collection procedures due to time constraints). Data from the remaining forty-six subjects (20 males) were retained for analysis; the ages of these participants ranged from 18–32 years (mean ± SD, 23.3±3.5 years).

### Experiment 1: Continuity/discontinuity perception

#### Stimuli

**Figure 1:**
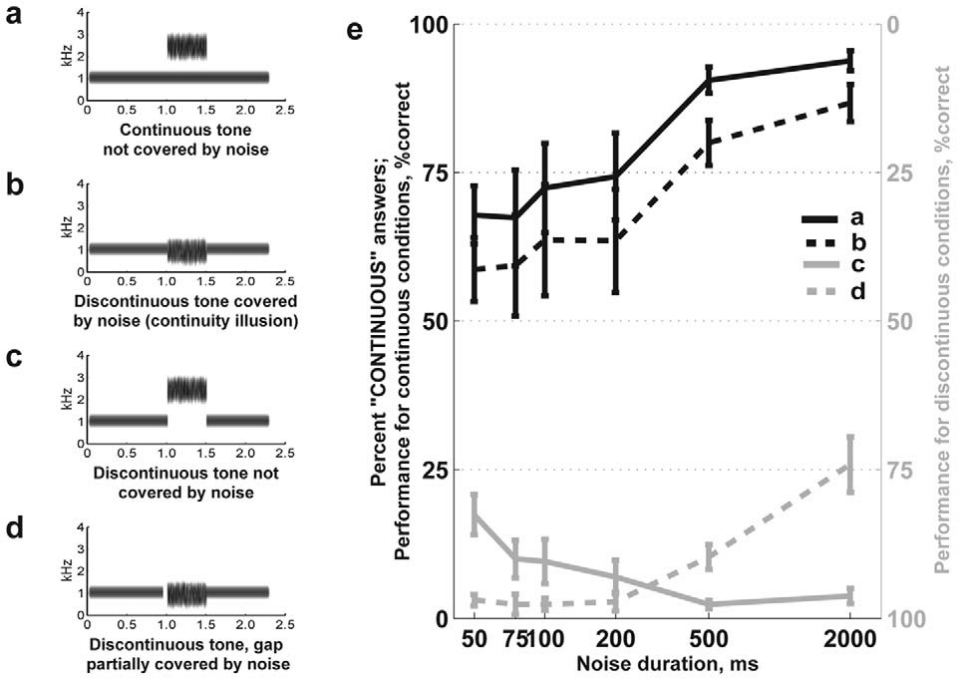
Psychophysical tests for perceptual continuity. **A–D**: Sound spectrograms of **s**timuli used in the four experimental conditions. In conditions B, C, and D, there is no tone physically present under the noise. The tone in condition B is often “illusorily” perceived as continuous. In condition D a gap between the tone and the noise starts 50 ms earlier than in C and B. The tone (1007 Hz) is separated from the lower edge of the noise in conditions A and C (2125 Hz) by more than one octave. **E**: Psychometric functions. See the legend on the right of panels a,b,c,d. The mean value ±1 SE is shown (n = 46 for noise durations of 50, 500, and 2000 ms, n = 18 for 75,100 and 200 ms). Combined data for pure tones and AM tones are reported here.

Target tones were combined with two kinds of noises (“masking noise” and “spectrally dissimilar noise”). The four conditions are illustrated in Figure 1. Fig. 1a and c represent cases of physically continuous (File S1) and discontinuous tones, respectively. In Fig. 1b, a masking noise occluded a gap in the tone, causing the subjective experience of the tone continuing through the noise (the auditory continuity illusion [5], [29], File S2). In a discontinuous condition (Fig. 1d) the tone ended 50 ms before the onset of the noise, breaking down this continuity illusion [4].

The noise always started 1000 ms after tone onset. The second portion of the tone after the noise ended was always 800 ms long. The noise durations (and, accordingly, the gap or tone-plus-noise “central part” durations) ranged from 50 ms to 2000 ms. Two different sets of noise durations were used in separate replications of the experiment: 50, 75, 100, 200, 500, 2000 ms in the first experimental run (18 participants) and 50, 500 or 2000 ms in the replication (another 28 participants). Noise samples were generated individually for each trial.

All target tones were either 1007 Hz pure tones or 1007 Hz amplitude-modulated tones (modulation frequency 40 Hz; modulation depth 0.9). The tone root-mean-square intensity was set to be 7dB lower than that of the noise, which in these experiments corresponded to a stimulus level of ∼60–70 dB SPL. All tones and noises had 7-ms cosine-square ramps at the sound edges.

Noise bursts were centered at 900 Hz (“masking noise”) or 2425 Hz (“spectrally dissimilar noise”), and had a bandwidth of 600 Hz. The lower edge of the higher-frequency noise burst was separated from the tone by more than one octave (about 6 equivalent rectangular bandwidths (ERBs) away [30]), to minimize the spectral overlap between the tone and noise.

Sounds were presented binaurally through E-A-RTONE 3A insert earphones with disposable tips (Aearo Corporation, Indianapolis, IN), with the noise level set at 68 dB HL. Hearing levels (HL) were utilized because of the disposable insert eartips used in this study. We referenced sound levels to the individual noise-detection thresholds of each participant. Noise-detection thresholds for the noise centered around 900 Hz were measured using a custom-built adaptive staircase procedure. The participants had comparable noise thresholds which were unrelated to their performance in the perceptual tasks utilized here (see Text S2).

#### Procedures

Participants were instructed to “ignore the noise, attend to the tone and report gaps or any kind of changes in the tone”. If they heard a gap or a change in the loudness of the tone they were asked to judge the tone as 'Discontinuous'. Examples and a practice session (with no feedback) were provided to facilitate attention to the tone during the noise.

For the first 10 minutes of the familiarization procedure, participants were asked to detect a decrease in intensity of an amplitude-modulated tone. The change could occur starting at 1000 ms after tone onset, and could last for 500 ms. In the second part of the familiarization procedure, also 10 minutes long, the task was the same, but a soft burst of noise similar in frequency to the tone appeared at the same time as the possible change in tone loudness.

The main experiment was divided into three 15-minute-long blocks with pauses between each block. Conditions and noise durations were randomized. Each participant performed 16 trials of each condition and noise duration.

#### Analysis

Group data and data from individual subjects were examined in each condition. The consistency of responses was estimated by calculating the probability of obtaining a given distribution of answers by chance using the cumulative binomial distribution function. If the probability of obtaining a given distribution by chance was smaller than 0.05, then a subject's answers were considered consistent. Otherwise, the subject's performance was considered to not differ from chance. A nonparametric measure of bias [31] was used to assess the possible differences in response criterion. A ‘Hit’ was scored when stimulus 1A or 1B was judged as continuous. A ‘false alarm’ was scored when stimulus 1C or 1D was judged as continuous. The bias B”*d* was calculated as follows:

### Experiment 2: EEG recordings under passive listening conditions

35 participants from the original subject pool (12 males, 5 left-handed) remained available to participate in this phase of the study. They were instructed to watch a silent movie and ignore experimental sounds that were presented binaurally through E-A-RTONE 3A insert earphones with disposable tips (Aearo Corporation, Indianapolis, IN). A 128-electrode ActiveOne data acquisition system (Biosemi B.V., Amsterdam, NL, www.biosemi.com) with sintered Ag-AgCl electrodes was used to record EEG and electro-oculogram (EOG) signals. The data was analyzed using EEGLab, Fieldtrip and custom-written software for MATLAB.

#### Stimuli

Two kinds of stimuli were presented: continuous tones accompanied by noises, and, in separate blocks, the same noise bursts presented alone, without the tone.

In the "tone + noise" blocks, stimuli were composed of a tone overlapped with a spectrally distant noise (Figure 1A, File S1) of two different durations, 50 ms or 1000 ms. These particular durations were chosen because the 50 ms noise burst caused a strong illusory auditory discontinuity in susceptible participants, whereas the 1000 ms noise did not. The noise was centered at 2425 Hz, had a bandwidth of 600 Hz (“spectrally dissimilar noise”), and appeared 1 second after tone onset. The tone was a 1007 Hz sinusoid, modulated in amplitude by a 40 Hz sinusoid. Intertrial intervals randomly varied from 900 to 1400 ms. Loudness of the tones was set 7 dB below the level of the noises, i.e. at 61 dB HL. The stimuli were presented in pseudorandom order during each 14.5-minute-long recording block.

In the "noise alone" blocks the noise bursts were presented in isolation, without the concurrent tone. All noises were 50 ms long. Two types of noises were presented: centered at 900 Hz (“masking noise”) or at 2425 Hz (“spectrally dissimilar noise”); both had a bandwidth of 600 Hz. The two noise types were equiprobable and were presented in pseudorandom order. Intertrial intervals ranged from 900 to 1400 ms. Independently-prepared noise samples were used for each trial. The stimuli were the same for all participants. Stimuli were generated using MATLAB (The MathWorks, Natick, MA) with a 44.1 kHz sampling rate.

#### Analysis

A subset of 64 electrodes evenly distributed across the scalp were used for all the analyses reported here to ease the computational load. An average reference, taken 200 ms before noise onset, was used. To avoid multiple comparison problems in the spatial domain, a single frontocentral electrode (Fz in the 10–20 coordinate system) was chosen *a-priori* for selection of time intervals of interest and for analyses, based on previous auditory EEG work [32]. This location was useful both for detecting components of evoked response with central topography, as well as other components with more frontal topography [33]. Three windows of interest were initially identified based on the morphology of waveform peaks (40–66 ms after noise onset (P_50_); 95–130 ms after noise onset (N_1_); 160–220 ms after noise onset (P_2_). The 270–350 ms after noise onset window was included in the analysis *a-posteriori*. To quantitatively characterize individual event-related potential (ERP) waveforms, the mean voltage of each individual's ERP at Fz in each integration window of interest was measured; all statistical tests used this single measure.

Significant correlations with perceptual performance were further assessed by examining the scalp EEG patterns to make sure these results were not artifacts confined to a single electrode. Spearman rank correlation coefficients [34] were used to quantify the relationship between ERP magnitude and perceptual performance for all 35 participants.

### Statistical analyses

Nonparametric statistics were used with both behavioral and electrophysiological data, because these data were not normally distributed. Paired-sample sign tests [34] or Wilcoxon matched-pair signed rank tests [35] were used to compare dependent samples, while Wilcoxon rank-sum tests [34] were used to compare independent samples. The Scheirer-Ray-Hare Two-way ANOVA [35] was used to assess cases with two variables that had multiple states. For single variables with multiple states, Kruskal-Wallis ANOVA [34] was used for independent samples and Friedman ANOVA for matched samples. Spearman rank correlation coefficients were used for all the correlation analyses. Benjamini-Hochberg false discovery rate [36] corrections were used to correct for multiple comparisons.

## Results

### Illusory discontinuity of tones

Forty-six normally-hearing participants were instructed to report whether the target tone was continuous or interrupted (that is, whether it contained any gap or change in the quality of the tone), and to ignore the noises that accompanied the tones. The stimuli included a continuous tone presented together with a higher-frequency noise burst more than one octave away (Fig. 1a), a discontinuous tone with spectrally overlapping noise (“illusory continuity”, Fig. 1b), and two matched discontinuous tone conditions (Fig. 1c, d).

There were no differences in perceptual responses between unmodulated and amplitude modulated tones (Scheirer-Ray-Hare Two-way ANOVA, p = 0.72), so responses to these two stimulus conditions were combined.

**Figure 2:**
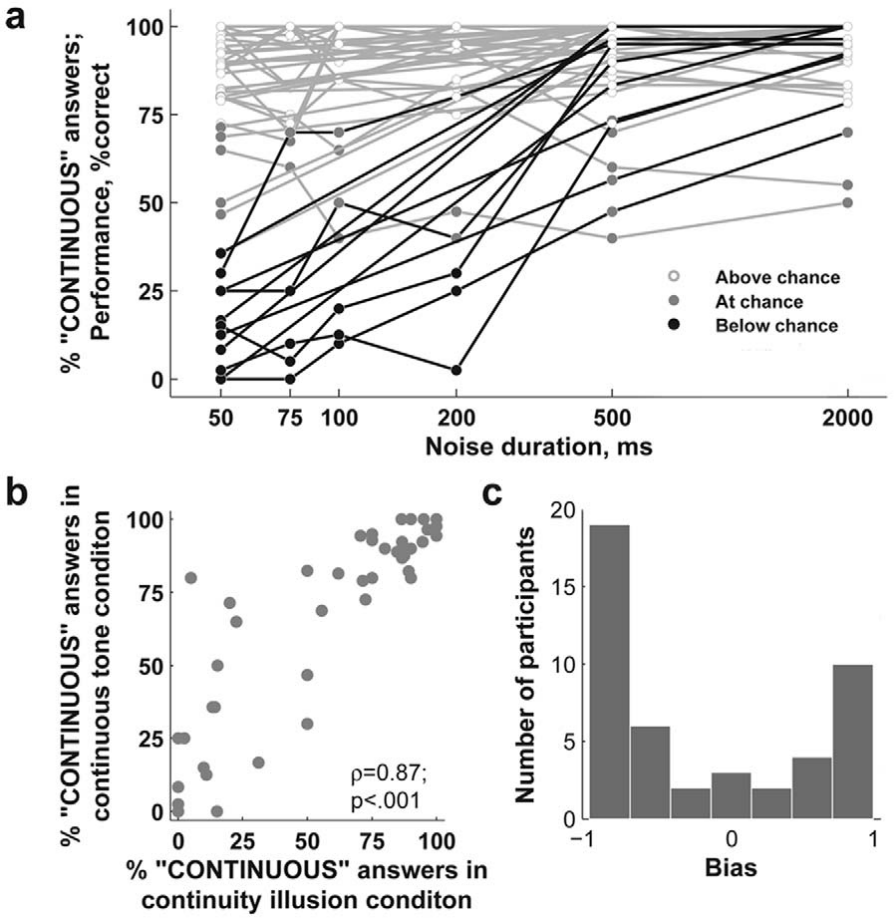
Illusory discontinuity. **a**. Individual performance in the continuous-tone-with spectrally-remote noise condition (Figure 1a). Subjects that exhibited illusory auditory discontinuity in trials with 50-ms noise bursts are drawn with black lines plotted in the foreground; all other subjects are plotted in gray. The color of the points indicates individual performance relative to chance levels defined by the binomial distribution. “Above chance” performance indicates a proportion of continuous responses significantly greater than chance at the p<0.05 level, while “below chance” indicates significantly fewer continuous responses than expected by chance (n = 46 for noise durations of 50, 500, and 2000 ms, n = 18 for 75,100 and 200 ms; individual chance levels are slightly different because of different numbers of trials.) **b**. Relationship between continuity responses in the continuous tone with spectrally remote noise condition (y-axis) and discontinuous tone covered by noise condition (x-axis). Each dot is a single subject; data come from 50 ms noise durations, n = 46. **c**. Distribution of individual response biases. ‘−1’ signifies a bias towards ‘discontinuous’ responses, and ‘1’ a bias towards ‘continuous’ responses. The data come from the same trials with 50-ms noises (n = 46).

We found a notable variability in individual performance in the continuous tone condition: it ranged from zero percent correct to 100% correct (Fig. 2a). Similar variability was seen in the continuity illusion condition, indicating that individual performance may have been influenced by response biases. Indeed, the distribution of response bias in trials with 50 ms noise was clearly bimodal (Fig. 2c; see Methods).

Participants who displayed a bias to continuity consistently gave “continuous” answers in the continuous tone condition and displayed a strong susceptibility to the continuity illusion (Figure 2b). In contrast, participants who exhibited a bias to discontinuity consistently reported continuous tones with remote noise bursts as discontinuous, and showed a weaker susceptibility to the continuity illusion.

We assessed reliability of individual performance independently for each of the four conditions by calculating the probability of obtaining the given answers by chance. A subject was considered to reliably perceive the sound as continuous or discontinuous if their performance differed from chance at the p<0.05 level specified by the binomial distribution (see Fig. 2a and Methods). Only 61% of all participants reliably judged the continuous tone (accompanied by short higher-frequency noises) as continuous. Eleven of the 46 participants (24%) reported these tones as discontinuous. These subjects are referred to as ‘susceptible to illusory discontinuity’. To characterize the strength of illusory discontinuity in the following analyses, we used the percent correct score in each condition rather than the bias measure, because the latter includes the effect of other factors which may or may not be related to the perceptual disruption of tone continuity.

### Susceptibility to illusory discontinuity and illusory continuity are negatively related

A strong negative relationship was found between the perceptual completion of obscured sounds, and the perception of physical continuity. The more reliably an individual judged the continuous tone as continuous, the greater was his or her susceptibility to the continuity illusion (Figure 2b, Spearman ρ = 0.87, N = 46, p<0.001). Therefore, the bias described above stemmed from consistent behavior in both the continuous tone with remote noise condition, and the continuity illusion condition. The strength of the continuity illusion increased with increasing noise duration (χ^2^ = 26.8, N = 46, p<0.0001, Friedman ANOVA), closely paralleling the decrease in illusory discontinuity with increasing noise duration (Figure 1e). Thirteen percent of all participants never consistently reported hearing the continuity illusion (their performance was either below chance or not significantly different from chance over all noise durations; see Figure 2b and Table S1). The present experiment, as well as previous experiments that reported relatively long durations for the continuity illusion, have all used band-passed noises rather than white noise [5], [20], [29]. Gaps longer than 2000 ms were not used in the present experiment; however, previous studies [29] suggest that the strength of the continuity illusion declines at longer noise durations. There was no relationship between thresholds for noise detection in quiet and the perception of auditory continuity or discontinuity (see Text S2).

### Neurophysiological differences between participants prone to illusory discontinuity and illusory continuity perception

We hypothesized that individual differences in perceiving illusory discontinuity could reflect differences in the neural processing of sound onsets. To examine neurophysiological differences related to behavioral performance, we recorded evoked electrical brain responses to the stimuli in a passive listening paradigm, while subjects' attention was captured by a silent movie.

The relationship between individual susceptibility to illusory discontinuity and electrophysiological responses was assessed using the stimuli that caused the strongest illusory discontinuity (a tone with by a spectrally remote 50 ms noise burst), presented in a passive listening context. Subjects were fitted with a 128-sensor electroencephalographic (EEG) array, and were exposed to the stimulus sounds in a comfortable position in a sound-attenuated chamber while watching an engaging silent movie of their choice. Thirty-five out of the 46 subjects who participated in Experiment 1 were able to participate in the EEG experiment; 6 reliably heard continuous tones as discontinuous, 9 had performed at chance levels with respect to their continuity perception, and 20 had reliably heard the continuous tones as continuous.

**Figure 3:**
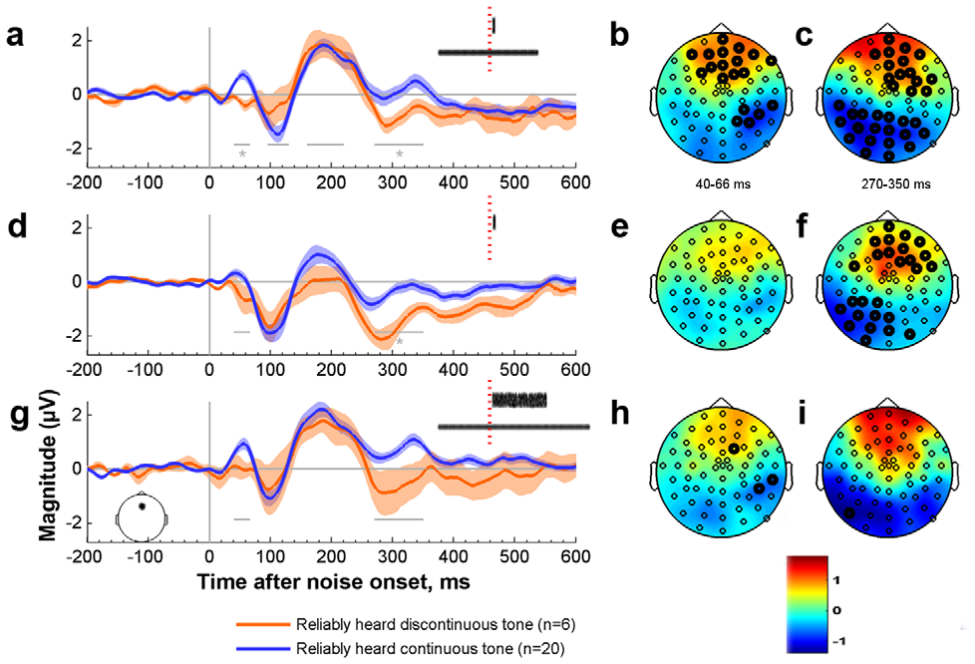
ERP aligned to the onset of noise bursts: **a,b,c,** 50-ms noise burst presented together with the tone; **d,e,f** 50-ms noise burst alone. **g,h,i** 1000-ms noises together with the tone (see also insets in panels a,d,g). A,d,g: Lines are group means and shaded areas are standard errors. Horizontal gray lines denote time windows of interest. Gray asterisks next to them indicate the significant correlation between magnitude and performance (test was done using all 35 participants, and not only the two groups of participants depicted here for clarity). Panels b,e,h refer to P50 component (40–66 ms after sound onset). Panels c,f,i refer to N270–350 component. In b,c,e,f,h,i color denotes the scalp distribution of the potential differences between participants that reliably heard the continuous tone as continuous and those that reliably heard it as discontinuous. Electrodes with nominally significant correlations between performance and voltage in all subjects (p<0.05, uncorrected) are highlighted with bold circles.

A significant correlation between psychophysical performance and the magnitude of EEG responses to noise bursts was found in two time windows out of the four that were selected for analysis (see Methods and gray asterisks in Figure 3a). There was a positive relationship between continuity perception and the magnitude of the P_50_ component (Figure 3a, 40–66 ms after noise onset, Spearman ρ = 0.47, n = 35, p = 0.018 (corrected for multiple comparisons) at the Fz electrode selected *a-priori* for analysis. Surrounding electrodes exhibited a similar pattern (bold dots in Figure 3b). The P_50_ component was absent in participants susceptible to illusory discontinuity (Figure 3a, red line, 40–66 ms after noise onset), whereas subjects who consistently heard these stimuli as continuous had a robust P_50_ response (Figure 3a, blue line, also Figure 3b: the difference between “continuous” and “discontinuous” groups is positive on frontal electrodes, as denoted by red color). Participants who perceived the tone as continuous also showed a significantly more positive integrated voltage of the N_270–350_ waveform peak between 270 to 350 ms after noise onset (Spearman ρ = 0.38, n = 35, p = 0.046, corrected for multiple comparisons, Figure 3a,c).

Response magnitudes at the P_50_ and the N_270–350_ time integration windows were moderately and significantly correlated within individual subjects (Spearman ρ = 0.35, p = 0.04 averaged across all conditions).

Responses to noise bursts were similar in “noise alone” and “tone plus noise” conditions; in both cases participants susceptible to illusory discontinuity showed smaller P_50_ magnitudes and a less positive N_270–350_ potential (compare Figures 3a and 3d). These differences were analyzed using (1) the correlation between performance and EEG voltage in the specific time windows, and (2) ANOVA. In the noise alone condition (Fig. 3d), only N_270–350_ showed a significant correlation with performance (indicated by a gray asterisk in panel d, ρ = .48, n = 35, p = 0.008). Unlike the P_50_ response when the tone was present, the P_50_ response in the noise alone condition showed no significant overall correlation between susceptibility to illusory discontinuity and response magnitude (Spearman ρ = 0.07, n = 35, p>0.9). However, in both of these time windows, nonparametric ANOVAs yielded significant main effects for subject performance (factors: condition (“noise alone” vs. “tone plus noise”) and subject performance (reliably perceive as continuous vs. discontinuous); P_50_: H = 25.8, df = 51, p = 0.044; N_270–350_: H = 31.9, df = 51, p = 0.019; both corrected for multiple comparisons). There was no significant effect of stimulus condition (P_50_: H = 2.6, df = 51, p>0.1; N_270–350_: H = 12.5, df = 51, p>0.1) and no significant interaction between subject performance and stimulus condition (H = 2.8 and 0.8, respectively, df = 51, p>0.1). This suggests that the pattern of responses was similar for the “tone plus noise” and the “noise alone” conditions, even though the latter condition had its strongest effect during the N_270–350_ window.

Illusory discontinuity was perceptually strongest at short noise durations and declined with longer noise durations (Figure 1e). Correspondingly, in the “tone plus long noise” condition there were no significant correlations between performance and ERP magnitude (Figure 3g, no gray asterisks). However, the tendency was the same: participants who showed low susceptibility to illusory discontinuity had larger P_50_ magnitudes and more positive N_270–350_ responses. We speculate that a long noise might initially act as a short one, but because of its duration, it is eventually segregated from the tone, resulting in no illusory discontinuity.

## Discussion

Sixty-one percent of the participants in the present study reliably heard a continuous tone accompanied by a brief noise burst more than one octave away as continuous, while 24% consistently judged this tone to be interrupted during the noise burst. Most susceptible participants described their sensations in terms of the tone actually containing a physical gap. However, it is possible that in participants with a milder subjective disruption of continuity, the behavior reflected perceptual uncertainty rather than the actual sensation of a tone being stopped during the noise. This perceptual uncertainty appeared to be the result of individual differences in sound processing, because the perceptual differences were correlated with brain response differences recorded in a passive listening paradigm. Recent work on the perception of illusory continuity in vowels has also suggested that such percepts operate independently of attentional state [37].

We hypothesize that individuals susceptible to illusory discontinuity lose the perceptual integrity of a continuous, pre-existing tone in the presence of a short-duration noise burst because their auditory system does not segregate, in a reliable and fast manner, the neural activity evoked by the noise burst and the activity representing the tone. In our experiment, longer noises caused less perceptual interference. Participants who judged the continuous tone as discontinuous also did not report continuity illusions under conditions where they are supposed to be strongest. This would make sense if the formation of independent perceptual streams is a prerequisite for the operation of perceptual processes that maintain the integrity of the separate streams [2], [9]. This is also in line with previous suggestions that both illusory continuity and streaming may depend on a common early mechanism that links together the parts of an auditory scene that are likely to be produced by the same sound sources [10], [38]. Preliminary data collected in a simple test of auditory perceptual streaming (see Text S1) also support a link between continuity perception and streaming; more exact perceptual characterizations will be required to rigorously test these ideas in future experiments.

Previous studies have consistently found that the continuity illusion is strongest in the case of noises that briefly interrupt a continuous tone [3], [29]; why might these studies not have detected the substantial proportion of participants who did not perceive illusory continuity when the interruptions were short? Most likely, the difference between the present and previous studies is due to differences in the participants subjective understanding of the task. The familiarization procedure and instructions used in the present study emphasized ANY possible changes in the perceptual characteristics of the tone during the noise, and thus permitted us to detect the breakdown of continuity perception in susceptible participants.

### Electrophysiological responses reflect individual differences in parsing auditory scenes

Dramatic differences in the perception of target sounds in the presence of maskers have long been known in normally-hearing listeners (e.g. [23]); physiological determinants of these individual performance differences have remained elusive. The present experiments found that individual susceptibility to illusory discontinuity was correlated with the magnitude of physiological responses to noise bursts when presented with or without a concurrent tone. Thus, we conclude that the reported EEG differences reflect the function of basic neural processes that modulate the susceptibility to the illusion.

Participants who perceived a tone with a short-duration remote-frequency noise burst as continuous showed larger onset responses 44–60 ms after noise onset. The P_50_ component (named for the latency of the response) is known to depend on the recent history of the stimulus [39]. Its origin and functional properties have been extensively studied, and weaker P_50_ suppression (altered “sensory gating” of repeated stimuli) has been reported in schizophrenic patients and their relatives [40]. P_50_ is thought to have multiple generators, including medial and intermediate levels of Heschl's gyrus (e.g., [41]–[45]), planum temporale [44], and the lateral surface of the STG [46]. The main generators of scalp-recorded P_50_ seem to originate from secondary auditory areas, mainly in the superior temporal gyrus [47]. Processes underlying P_50_ generation and its habituation are presumably important for detecting novelty in the environment, and thus contribute to auditory scene analysis.

At least two previous studies, using other methods, showed that stronger stimulus-evoked activity is beneficial for auditory perceptual detection and identification. BOLD signals in anterolateral portions of Heschl's gyrus and superior temporal gyrus correlated with individual accuracy of sound identification in noise, whereas signals in inferior frontal regions correlated with the subjects' reaction times [48]. Elhilali et al. [17] examined steady-state responses to a train of sounds when subjects attended to those sounds, or to a masker presented simultaneously. The results showed a link between the neural representation of the attended target and its perceptual detectability over time. The present data add to this line of research by showing that the representation of a masker stimulus (noise) is also important for the perception of target sounds, presumably because it contributes to the segregation of the mixture.

The moderate within-subject correlation between the magnitude of the early P_50_ and late N_270–350_ potentials seen in the present study suggests that inter-subject variability in later stages of processing may partially depend on the nature of early responses. Some attributes of the evoked N_270–350_ potential difference examined here appear to be similar to those of the previously-described P_3a_ potential, which has been argued to reflect frontal lobe-mediated involuntary attention when a novel stimulus appears in a scene [49]. Unlike the P_300_, usually seen in response to attended novel objects or sounds, P_3a_ may also reflect irrelevant sound changes, and appears in passive recording situations in about 15% of recorded participants 49]. It remains to be seen whether the psychophysical and electrophysiological changes found in our paradigm would be consistent with individual measures of P_50_ suppression and P_3a_ obtained in standard paradigms.

We speculate that the observed perceptual differences seen here are part of people's normal variation in auditory abilities; however, this underlying variation might importantly contribute to reductions in an individual's quality of life following even small amounts of hearing loss, by increasing the difficulty of extracting meaningful signals from noisy environments.

## Supporting Information

File S1 Example of Discontinuity illusion stimulus: Continuous tone with spectrally remote noise. The tone sounds discontinuous to some listeners.
(WAV)

File S2 Example of Continuity illusion stimulus (noise duration 50 ms).
(WAV)

Table S1 Individual performance in trials with 50 ms-long noise.
(DOC)

Text S1 Preliminary Data Streaming and illusory discontinuity are related.
(DOC)

Text S2 No relationship between noise detection thresholds and continuity/discontinuity perception.
(DOC)
