# Supplementary material for: Individual Differences in Sound-in-Noise Perception Are Related to the Strength of Short-Latency Neural Responses to Noise
Source: PLoS One. 2011 Feb 28;6(2):e17266. doi: 10.1371/journal.pone.0017266 (PMC3046163; doi:10.1371/journal.pone.0017266)
Supplement: Table S1 — Individual performance in trials with 50 ms-long noise. (DOC) [file pone.0017266.s003.doc]

**Supplementary table S1**

**Table S1** Individual performance in trials with 50ms-long noise.

|  |  | **Number of participants that**  **exhibit this behavioral pattern** |
| --- | --- | --- |
| **Physical continuity** | Perform below chance  (Hear gaps reliably in a continuous tone) | 11 (24%) |
|  | Perform above chance  (Reliably hear continuous tones as continuous) | 28 (61%) |
|  | Variable performance  (not different from chance) | 7 (15%) |
| **Illusory continuity** | Perform below chance  (do not reliably hear the continuity illusion) | 13 (28.3%) |
|  | Reliably perceive the continuity illusion | 25 (54.3%) |
|  | Variable performance  (not different from chance) | 8 (17.4%) |
|  | Total number of participants | 46 |
