# Supplementary material for: Individual Differences in Sound-in-Noise Perception Are Related to the Strength of Short-Latency Neural Responses to Noise
Source: PLoS One. 2011 Feb 28;6(2):e17266. doi: 10.1371/journal.pone.0017266 (PMC3046163; doi:10.1371/journal.pone.0017266)
Supplement: Text S1 — Preliminary Data Streaming and illusory discontinuity are related. (DOC) [file pone.0017266.s004.doc]

### Supplementary text S4

**Preliminary Data: Streaming and illusory discontinuity are related**

To test the hypothesis that illusory discontinuity may reflect some kind of perceptual interference between the tone and the noise that makes it more difficult to segregate them quickly, we compared susceptibility to illusory discontinuity with pitch-based sound segregation scores, measured in a standard auditory streaming-by-frequency-separation paradigm (Bregman, 1990).

**Stimuli**. Individual streaming abilities were assessed using a set of pure tones, alternating in frequency in a triplet rhythmic pattern (ABA ABA…), based on the procedure described by van Noorden (1975). Depending on the frequency separation between the tones (Δf), the sequence of tones could be heard as a single melodic stream or as 2 different interleaved streams with distinct rhythms. The frequency separation ranged from 0.6 to 16 semitones. Each tone lasted 150 ms; inter-tone intervals were 150 ms. The overall duration of the sequence was 4 seconds (shorter than in previous studies, in order to specifically emphasize the process of the build-up of streaming). The tones were centered around 1,007 Hz and were of the same intensity as in Experiment 1.

**Procedures.** On each trial, the participants were asked to estimate the degree of perceptual separation of lower and higher tones in the melody on a subjective scale from 1 to 5. 'One' meant that a single melody of alternating tones was heard throughout the trial, and 'five' corresponded to a strong perceptual separation into one lower and one higher pattern starting from the beginning of the sequence. 'Three' was used to indicate trials on which the low and high patterns separated during the course of a trial. Examples of highly grouped and highly separated sound sequences, as well as familiarization trials (n=20) were provided. Each participant performed 70 test trials.

**Analysis.**  In the streaming test, all participants heard tones as a single melody at the smallest frequency separation (mean score ± SE 1.3±0.1). At the largest frequency separation, all participants heard the tone sequence as two distinct melodies (mean score ± SE 4.8±0.05). A single measure of streaming for each subject was calculated by averaging the data from intermediate frequency separations of 2-7 semitones, where judgements varied considerably among participants. As a measure of susceptibility to noise (disruption of perceived continuity) we took performance in the continuous-tone-plus-50-ms-spectrally-remote-noise condition from Experiment 1, since the shortest noise duration caused the strongest illusory discontinuity.

**Results:** Twenty-eight of the 46 participants were tested using an ABA…ABA auditory streaming task (see Methods); 6 reliably heard continuous tones as discontinuous, 9 had performed at chance levels with respect to their continuity perception, and 12 had reliably heard the continuous tones as continuous. Depending on the pitch distance between the A and B tones, the sequence could be perceived as a single alternating melody or two interleaved melodies that differ in pitch and rhythm (Bregman, 1990). As expected, there was considerable individual variability in streaming scores: the ABA sequences with intermediate frequency separation were perceived as grouped by some participants and as segregated by others. A moderate, statistically-significant negative correlation was obtained between susceptibility to illusory discontinuity and streaming scores (Spearman ρ = -0.39, N=28, p= 0.039). Participants most susceptible to illusory discontinuity tended to group the tones in a single stream, whereas the least susceptible participants were more likely to segregate tones into two distinct streams.

### No relationship between noise detection thresholds and continuity / discontinuity perception

**Procedures**: Noise detection thresholds were measured using custom-written programs that employed two types of stimulus sounds from Experiment 1 (500-ms-long band-pass noises either from 600 to 1200 Hz or from 2100 to 2700 Hz). The participants’ task was to detect the presence or absence of the sound during a given time interval. An adaptive staircase started from a clearly audible sound level and adjusted the amplitude of the noise in a 2 up, 1 down procedure, with step size decreasing over time (step up/step down ratio = .9). The procedure was stopped after 14 reversals, and the noise detection threshold was calculated as the mean value over the last 10 reversals. These thresholds were determined successively for each ear and noise type in random order. Noise detection thresholds were analyzed for the 28 subjects who participated in the preliminary streaming experiment. The mean RMS voltage of the noise stimulus at threshold for the population of 28 participants was 0.058 + 0.091 Volts.

**Results**: No significant relationships were found between tone detection thresholds in noise and continuity/discontinuity perception. The participants’ detection thresholds for band-passed noise in quiet did not correlate with either the percentage of “continuous” answers to the continuous-tone-with-remote-frequency-noise stimulus (Spearman ρ =-0.21, N = 28, p=0.29), nor with individual perceptual streaming scores (ρ =-0.041, N = 28, p=0.84). This demonstrates that perceptual restoration and streaming differences in these participants were not simply the result of differing thresholds for noise detection.
