# Supplementary material for: Individual Differences in Sound-in-Noise Perception Are Related to the Strength of Short-Latency Neural Responses to Noise
Source: PLoS One. 2011 Feb 28;6(2):e17266. doi: 10.1371/journal.pone.0017266 (PMC3046163; doi:10.1371/journal.pone.0017266)
Supplement: Text S2 — No relationship between noise detection thresholds and continuity/discontinuity perception. (DOC) [file pone.0017266.s005.doc]

### Supplementary text S5

### No relationship between noise detection thresholds and continuity / discontinuity perception

**Procedures**: Noise detection thresholds were measured using custom-written programs that employed two types of stimulus sounds from Experiment 1 (500-ms-long band-pass noises either from 600 to 1200 Hz or from 2100 to 2700 Hz). The participants’ task was to detect the presence or absence of the sound during a given time interval. An adaptive staircase started from a clearly audible sound level and adjusted the amplitude of the noise in a 2 up, 1 down procedure, with step size decreasing over time (step up/step down ratio = .9). The procedure was stopped after 14 reversals, and the noise detection threshold was calculated as the mean value over the last 10 reversals. These thresholds were determined successively for each ear and noise type in random order. Noise detection thresholds were analyzed for the 28 subjects who participated in the preliminary streaming experiment. The mean RMS voltage of the noise stimulus at threshold for the population of 28 participants was 0.058 + 0.091 Volts.

**Results**: No significant relationships were found between tone detection thresholds in noise and continuity/discontinuity perception. The participants’ detection thresholds for band-passed noise in quiet did not correlate with either the percentage of “continuous” answers to the continuous-tone-with-remote-frequency-noise stimulus (Spearman ρ =-0.21, N = 28, p=0.29), nor with individual perceptual streaming scores (ρ =-0.041, N = 28, p=0.84). This demonstrates that perceptual restoration and streaming differences in these participants were not simply the result of differing thresholds for noise detection.
